# Local melting to design strong and plastically deformable bulk metallic glass composites

**DOI:** 10.1038/srep42518

**Published:** 2017-02-13

**Authors:** Yue-Sheng Qin, Xiao-Liang Han, Kai-Kai Song, Yu-Hao Tian, Chuan-Xiao Peng, Li Wang, Bao-An Sun, Gang Wang, Ivan Kaban, Jürgen Eckert

**Affiliations:** 1 School of Mechanical, Electrical & Information Engineering, Shandong University (Weihai), Wenhua Xilu 180, 264209 Weihai, P.R. China; 2 Centre for Advanced Structural Materials, Department of Mechanical and Biomedical Engineering, City University of Hong Kong, 999077 Hong Kong SAR, P.R. China; 3 Laboratory for Microstructures, Shanghai University, 200444 Shanghai, P.R. China; 4 IFW Dresden, Institute for Complex Materials, Helmholtzstraße 20, 01069 Dresden, Germany; 5 Erich Schmid Institute of Materials Science, Austrian Academy of Sciences, Jahnstraße 12, A-8700 Leoben, Austria; 6 Department Materials Physics, Montanuniversität Leoben, Jahnstraße 12, A-8700 Leoben, Austria

## Abstract

Recently, CuZr-based bulk metallic glass (BMG) composites reinforced by the TRIP (transformation-induced plasticity) effect have been explored in attempt to accomplish an optimal of trade-off between strength and ductility. However, the design of such BMG composites with advanced mechanical properties still remains a big challenge for materials engineering. In this work, we proposed a technique of instantaneously and locally arc-melting BMG plate to artificially induce the precipitation of B2 crystals in the glassy matrix and then to tune mechanical properties. Through adjusting local melting process parameters (i.e. input powers, local melting positions, and distances between the electrode and amorphous plate), the size, volume fraction, and distribution of B2 crystals were well tailored and the corresponding formation mechanism was clearly clarified. The resultant BMG composites exhibit large compressive plasticity and high strength together with obvious work-hardening ability. This compelling approach could be of great significance for the steady development of metastable CuZr-based alloys with excellent mechanical properties.

Recent breakthrough discovery of CuZr-based bulk metallic glass (BMG) composites fabricated by introducing the transformation-induced plasticity (TRIP) effect opens up a new perspective for the design of ductile BMG composites^1^,^2^,^3^,^4^,^5^,^6^,^7^,^8^,^9^,^10^,^11^. BMG composites with shape memory B2 CuZr phase embedding into the glassy matrix exhibit outstanding strength and tensile plasticity without significant trade-off but with obvious work hardening^1^,^2^,^3^,^4^,^5^,^6^,^7^,^8^,^9^,^10^,^11^. During deformation, B2 CuZr crystals in the glassy matrix can transform into martensites and simultaneously induce the formation of multiple shear bands, resulting in superior mechanical properties of CuZr-based alloys^1^,^2^,^3^,^4^,^5^,^6^,^7^,^8^,^9^,^10^,^11^. Yet, the mechanical properties of such TRIP-reinforced BMG composites strongly depends on their microstructural features such as the volume fraction, particle size, and inter-particle spacing of crystalline constituents in the glassy matrix, and both the design and control of these features still present a major challenge^1^,^2^,^3^,^4^,^5^,^6^,^7^,^8^,^9^,^10^,^11^.

Until now, only a limited number of fabrication methods have been developed to control the precipitation of B2 CuZr crystals in the glassy matrix, which include optimizing CuZr-based glass-forming compositions^4^,^5^,^6^,^7^,^8^,^12^,^13^, re-melting master alloys before rapid solidification^14^, adjusting applied cooling rates^12^,^15^,^16^, controlling casting parameters^17^, and introducing inoculants for heterogeneous nucleation of B2 phase^18^,^19^. Recently, by applying rapidly heating Cu-Zr-Al amorphous ribbons, the high-temperature B2 CuZr microcrystals in glassy matrix were induced and kept to room temperature^20^, leading to the formation of ductile BMG composites. Hence, by partially and rapidly crystallizing BMGs or controlling rapid solidification of melts, B2 CuZr crystals can be formed in the glassy matrix in CuZr-based alloys. Even so, it is still difficult to locally and precisely control the formation of B2 CuZr crystals and simultaneously maintain the matrix in an amorphous state.

In order to achieve such a goal, we proposed a method of the instantaneous and local heat treatment on BMGs by electric arc melting. When a BMG is locally and rapidly heated and even melted, the remained matrix is still amorphous by applying a cooling system. Furthermore, it is easy to control the spatial distribution of crystals in the glassy matrix by changing the position of the arc electrode, and to fulfil one of the main requirements for the composites^4^,^5^,^6^,^7^,^8^,^9^,^10^,^11^,^12^,^13^,^14^,^15^,^16^,^17^,^18^,^19^,^20^. In this work, this strategy is applied to control the local precipitation of the B2 phase in CuZr-based BMG. Furthermore, the mechanical properties of the obtained CuZr-based BMG composites are investigated and their underlying deformation mechanism is discussed.

## Results and Discussion

### Microstructural evolutions before and after local melting

**Figure 1:**
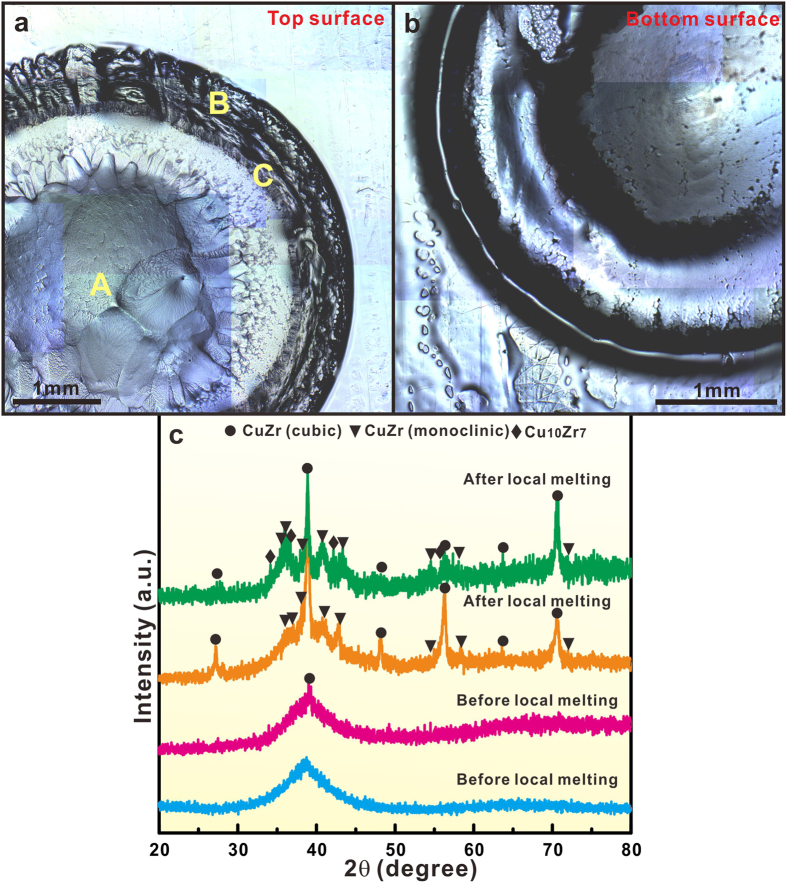
OM images of the top (**a**) and the bottom surface (**b**) of a Cu_47_Zr_47_Al_6_ plate after local melting; (**c**) –XRD patterns for the samples before (from different as-cast BMG plates) and after local melting under a melting current/voltage of 30 ± 2 A/14 ± 1 V and 50 ± 2 A/12 ± 1 V, respectively.

The OM images taken from the top and bottom surfaces of a Cu_47_Zr_47_Al_6_ plate after local melting with an input power of 604 ± 124 W are shown in Fig. 1a and b. The center of the top surface is obviously concave while that of the bottom surface is gibbous. Radially directed coarse dendrites are seen at the top surface (Fig. S1a), whereas much finer dendrites are observed at the bottom surface (Fig. S1b). Similar to the arc welding^21^,^22^, the arc-affected regions in the glassy matrix contain three zones, as marked in Fig. 1a: the molten or fusion zone (A), heat-affected zone (B), and narrow fusion boundary zone (C). The XRD patterns of the samples before and after local arc melting are plotted in Fig. 1c. The as-cast plates were virtually amorphous, although a small amount of B2 CuZr crystals precipitate in the glassy matrix for some samples due to its relatively large dimension. Since the local melting regions are very distinguished, the presence of such a small amount of B2 crystals cannot affect the validity of the present investigation. The XRD patterns of the samples after local arc melting reveal that a large amount of B2 crystals together with a little CuZr martensites form in the glassy matrix. With increasing the input power during local melting from 422 ± 58 W to 600 ± 124 W (Fig. 1c), the volume fraction of crystals in the glassy matrix also increases. Beside the formation of B2 CuZr and CuZr martensites, some Cu_10_Zr_7_ crystals are also observed for the input power of 604 ± 124 W during local melting.

**Figure 2:**
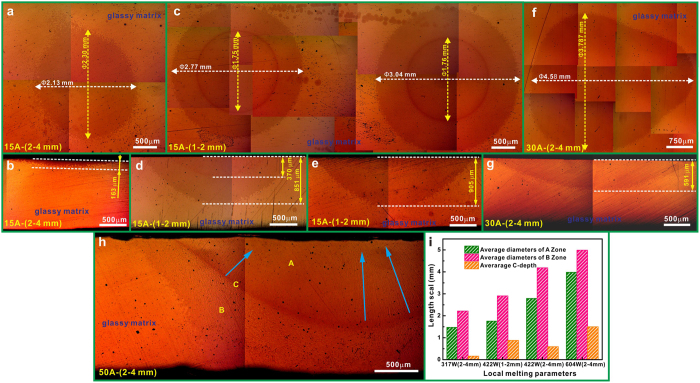
OM images of the top parts and the longitudinal sections of the samples after local melting applying different electric arc currents and DTC distances: (**a**–**b**) 15 A and 2–4 mm, (**c**–**e**) 15 A and 1–2 mm, (**f–g**) 30 A and 2–4 mm, (**h**) 50 A and 2–4 mm.

In the next step, the influence of the electric arc parameters on the formation of B2 phase was studied. For this, local melting of CuZrAl amorphous plates was performed by applying different input powers (i.e. 317 ± 57 W, 422 ± 58 W, and 604 ± 124 W) and changing distances between the tungsten electrode and the CuZrAl glassy plates (i.e. 1–2 mm and 2–4 mm, respectively). As shown in Fig. 2, the local crystallization in the glassy matrix occurs for all samples after local melting. At a constant electrode-to-plate distance, both the area of the crystallized region (measured at the surface of the plate) and the corresponding crystallized depth (C-depth) gradually increase with increasing electric arc current (Fig. 2). At a constant electrode-to-plate distance of about 2–4 mm (Fig. 2a, b, f, and g), the average diameter of the crystallized region increases from 2.2 ± 0.2 mm to 4.2 ± 0.4 mm, and the average C-depth increases from 163 ± 5 μm to 591 ± 5 μm when the input powers during local melting changes from 317 ± 57 W to 422 ± 58 W. When the electrode-to-plate distance decreases from about 2–4 mm to 1–2 mm under a constant input power of 317 ± 57 W, the average diameter of the crystallized spot increases from 2.2 ± 0.2 mm to 2.9 ± 0.2 mm and the C-depth increases from 163 ± 5 μm to 878 ± 27 μm, respectively. Finally, the C-depth approaches to the thickness of the Cu-Zr-Al plate itself, i.e. 1.5 mm, for the input power of 604 ± 124 W. All in all, as shown in Fig. 2i, the average diameters of the zones A and B can be enhanced by increasing the input powers during local melting. The average C-depth can be adjusted by changing the input power and the electrode-to-plate distance, respectively. Beside the geometric size of the locally molten and crystallized regions, it is extremely important to control their spatial distribution over the whole glassy matrix for obtaining the composites. This is rather simply achievable in the present method by moving the tungsten electrode to a desirable place as demonstrated in Fig. 2c for two spots produced at the same conditions. It is clearly seen that the crystallized surface areas and, respectively, the C-depth for both spots fabricated at the same conditions are very similar.

### Formation mechanism of B2 CuZr crystals during local melting

**Figure 3:**
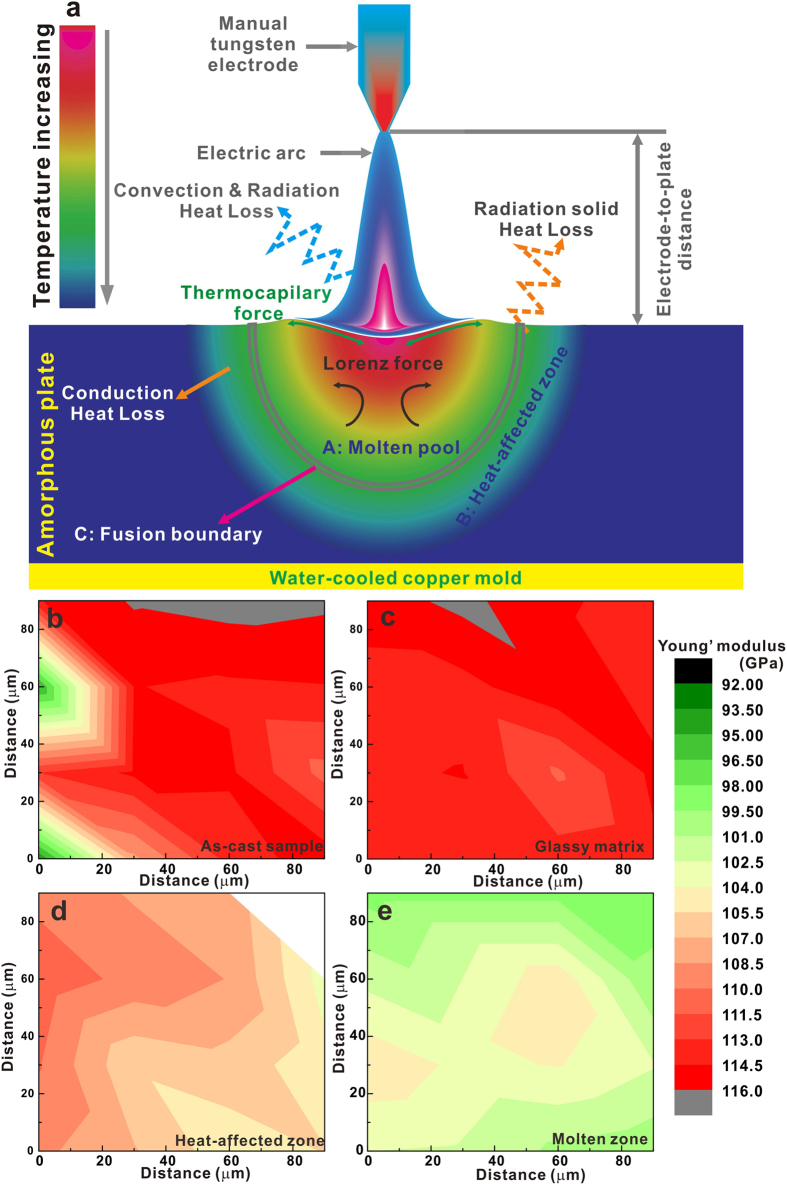
Schematic of the local electric arc melting (**a**) and distribution of Young’s modulus in Cu_47_Zr_47_Al_6_ plates: as-cast (**b**) and after electric-arc treatment for the glassy matrix (**c)**, heat-affected zone (**d**) and molten zone (**e**).

The electric arc local melting process is schematically illustrated in Fig. 3a. Once the electric arc is induced to the amorphous plate, it melts locally. The generated heat is dissipated by the convection, radiation, and heating the surrounding glassy matrix, respectively^21^,^22^,^23^,^24^,^25^,^26^. A part of the generated heat would be consumed the neighbouring argon atmosphere in the convection and radiation manners (see the blue line in Fig. 3a). However, most of the generated heat can be dissipated by the material itself in a conduction way during local melting, while the heated material itself also can loss some heat in a radiation manner from the surface (see the orange line in Fig. 3a). The surface of the melt is deformed due to the induced arc pressure (Fig. 3a)^21^,^22^,^23^,^24^,^25^,^26^, which causes that the final surface topology is not flat (Fig. 1a and b). Such a surface topography might result in the formation of residual stresses, negatively affecting the fabricated composite. However, this layer can be removed by polishing. Moreover, the Lorenz force induced by the electric arc can also drive the flow of the melt^21^,^22^,^23^,^24^,^25^,^26^. Previous experimental and simulation results have shown that the temperature field of a molten pool and the liquid metal in the molten pool exhibit a radial gradient^27^,^28^,^29^, resulting in a similar crystallization direction (blue arrows in Fig. 2(h)). Usually, the maximum temperature of the molten pool is larger than 1500 K^21–29^. This is certainly higher than the onset temperature and the final temperature of the melting for Cu_47_Zr_47_Al_6_ BMG which are 1150 ± 2 K and 1174 ± 2 K, respectively^9^. After removing the electric arc molten pool is rapidly quenched due to its small dimensions and cooling the plate by supporting water-cooled cooper block. Although the present cooling rate is higher than that of about 4 K/s reported for arc melting^30^, it is probably somewhat lower than the quenching speed of 200–770 K/s required preventing decomposition of the high-temperature B2 CuZr phase into the stable CuZr_2_ and Cu_10_Zr_7_ phases^31^,^32^. Therefore, a small amount of Cu_10_Zr_7_ crystals is formed under a high arc melting current. Furthermore, CuZr martensities are induced within B2 crystals due to the thermal stress during quenching^10^,^33^. Naturally, the present cooling rate is not sufficient to quench the melts into a fully amorphous state, but this is also not aimed.

The neighboring regions of the molten zone A are also heated mainly by the heat conduction in the metallic plate^27^,^28^,^29^. It has been demonstrated that the minimum and maximum temperatures of the heat-affected zone B can be at least 800 K and 1773 K^25^,^29^,^34^, respectively. The whole arc melting process is finished within 1 s, implying that the applied heating rates should be higher than 800 K/s. It is known that the glass transition temperature (*T*_*g*_) and the crystallization temperature shift to higher temperatures with increasing heating rate^35^. At a heating rate of 20 K/min, the B2 CuZr crystals precipitate at 1065 ± 2 K in the Cu_47_Zr_47_Al_6_ BMG^9^, and the crystallization temperature possibly increases by about 30 K at a heating rate of 2400 K/s^36^. A cooling rate of 200–770 K/s is required to suppress the decomposition of the high-temperature B2 CuZr phase^31^,^32^. As there is still a little amorphous phase along with major B2 CuZr crystals in the zone B, the applied cooling rate could be estimated to be between 770–1000 K/s here.

**Figure 4:**
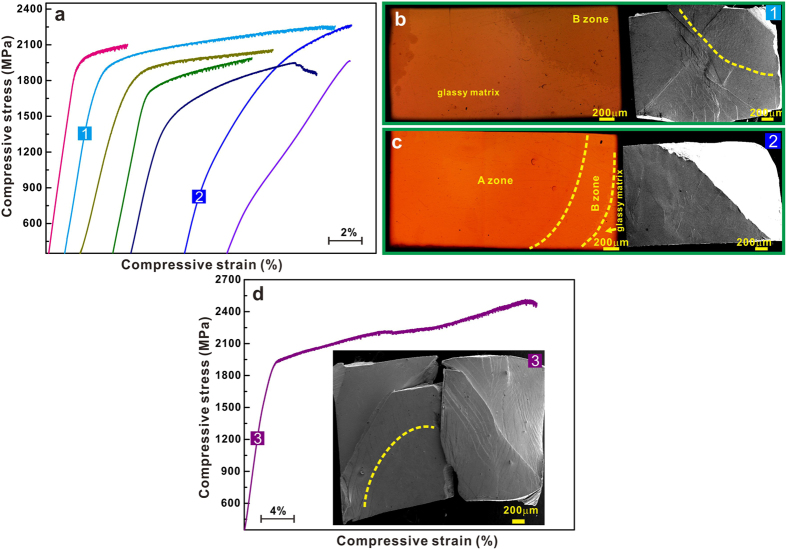
(**a**) Compressive stress-strain curves for the Cu_47_Zr_47_Al_6_ plates after local melting, showing enhanced plasticity with increasing volume fraction of B2 CuZr phase; The first (left) curve corresponds to the as-cast glassy plate; the next curves are measured on the samples after local arc melting (the crystalline volume fraction increases from the left to right). OM and SEM images for the samples 1 (**b**) and 2 (**c**) before and after deformation, respectively. (**d**) Stress-strain curve measured on a composite showing a high yield stress as well as remarkable plasticity (inset: the lateral surface for the sample 3 after deformation).

The glassy matrix neighboring to the B zone was also rapidly heated. The velocity of the heat transfer *v* can be estimated from the formula *Q* = *vρc*_*p*_Δ*T*, where *Q* is the applied heat flux during arc melting, *ρ* is the density and *c*_*p*_ is the heat capacity at constant pressure for the treated sample, and Δ*T* is the temperature gradient^37^. The power density by arc melting can be computed as the power entering the surface divided by the surface area^21^. In our case, the power applied on the surface is roughly estimated to be of about 300–600 W and the surface area between about 2.1 × 10^–5^ m^2^ and 3.8 × 10^–6^ m^2^ (Figs 1,3 and 4). The corresponding power density of about 2.9 × 10^7^–7.9 × 10^7^ W·m^−2^ is consistent with previous reports^21^. The density and the heat capacity for the Cu-Zr-Al BMGs were determined to be 7.0–7.4 × 10^3^ kg·m^−3^ and 400–420 J·Kg^−1^·K^−1^, respectively^38^,^39^. The Δ*T* is roughly estimated to be ~2000 K. With these values, the velocity of the heat transfer *v* is calculated to be about 4.7–14 mm/s, implying that the glassy matrix around the zone B is also rapidly heated by local arc melting. However, as the BMG plate is attached to the water-cooled copper block, the cooling rate is also very high for the area around zone B. Therefore, it still remains in an amorphous state.

### Mechanical properties of CuZr-based BMG composites fabricated by local melting

In order to investigate the mechanical properties of the BMG composites fabricated by local melting, the Vickers hardness, nanoindentation, and compression tests were conducted, respectively. The nanoindentation tests (Fig. 3) showed a very good correlation of the Vickers hardness and Young’s modulus with microstructures of the locally-melt plate. The HV_200g/5s_ values of the molten zone, heat-affected zone and glassy matrix are 375 ± 11 HV, 470 ± 14 HV and 493 ± 10 HV, respectively. The two-dimensional maps of the Young’s modulus distribution in the plates before and after local melting are also presented in Fig. 3b–e. The Young’s modulus is the lowest in the zone A where mainly B2 CuZr phase exists. It is larger in the zone B, implying that some amorphous phase could still exist. It is interesting that the distribution of the Young’s modulus for the glassy matrix of an electric arc treated plate is more uniform than that for the as-cast BMG plates (compare Fig. 3b,c). Since the nanoindentation test reflects properties of small nanometer-size volumes^40^, Fig. 3c implies that the structure of the glassy matrix becomes more homogenous after local melting compared to that of as-cast plate. This finding is consistent with previous results^41^,^42^ which showed that annealing of BMGs below *T*_*g*_ could degenerate structural inhomogeneities.

**Figure 5:**
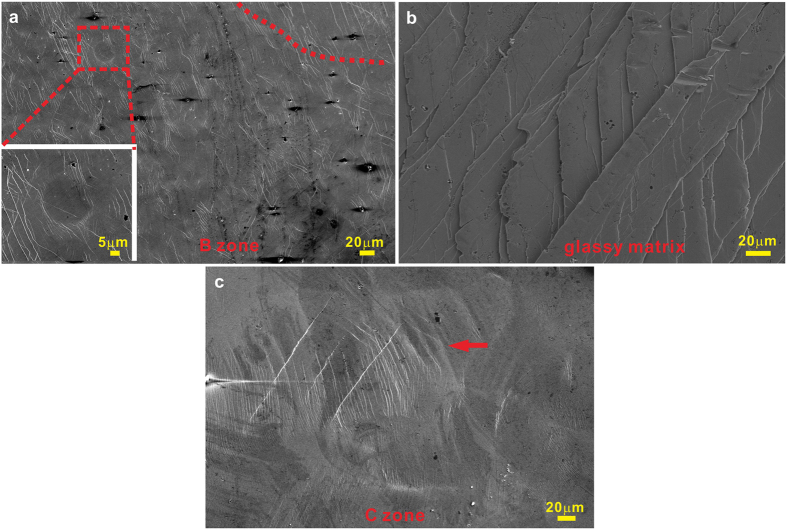
Lateral morphologies of (**a**) the heat-affected zone, (**b**) the glassy matrix, (**c**) the mixed zone consisting of the heat-affected zone and the molten zone for the locally melting samples after deformation.

The results of uniaxial compression tests carried out at room temperature on the Cu_47_Zr_47_Al_6_ plates before and after local arc melting (cut from the zones B and C) are presented in Fig. 4a and b. Compared with as-cast BMG, the BMG matrix composites show a lower yield strength but a better plasticity. The yield strength is decreased and plasticity is enhanced with increasing crystalline volume fraction (Fig. 4a). Previous studies have shown that the plastic strain together with a yield strength larger than 1650 MPa is usually up to 14% for CuZr-based BMG composites at room temperature^4^,^5^,^7^,^8^,^12^,^13^,^14^,^15^,^16^,^17^,^18^,^19^,^20^. Our results demonstrate that a significantly higher plastic strain (>14%) without obviously fracture and a yield strength of 1700 ± 10 MPa (see the sample 1 in Fig. 4a) when the volume fraction and spatial distribution of B2 crystals are properly controlled. In our cases, even though the OM image (the top surface of the sample 1 in Fig. 4b) suggests that the crystalline fraction might be more than 25 vol.%, the actual crystalline volume fraction is supposedly less than 15 vol.% since the corresponding C-depth is smaller than 1 mm and the zone B also contains a little amorphous phase (Fig. 4b). Therefore, a higher yield strength of 1700 ± 10 MPa can be observed in the sample 1. Besides, the deformed sample bulges in the middle instead of partially contacting the machine crosshead (Fig. 4b). The volume fraction of B2 crystals seems to decrease significantly in the sample 2 (Fig. 4a), which was mainly cut from the zones A and B (the yellow lines in the inset in Fig. 4c). However, the corresponding crystallized depth is less than 1.5 mm, and the region underneath the surface of the sample 2 still contains amorphous phase, leading to a yield strength of 1080 ± 10 MPa. It is worth noting that a large plastic strain (>33%) together with a high yield strength is also obtained when some B2 CuZr crystals are formed near the side of the intermediate region of the sample 3 (the yellow line in the inset in Fig. 4d). Furthermore, even a higher yield strength of 1840 ± 10 MPa was obtained in the sample 3 since its crystalline volume fraction is far less than 15 vol.% due to a limited C-depth. In such a case, the distortion of the sample in the part is induced due to a partial contract between the machine crosshead and the deformed part, being similar with some BMGs with super ductility during compression^43^. Recently, our group has studies the correlation between the volume fraction, spatial distribution, and size of B2 CuZr crystals and the fracture strains for CuZr-based BMGs composites^10^,^17^. The fracture strain of CuZr-based BMG composites can be well described by the equation:


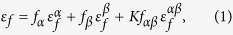


where *f*_*α*_, *f*_*β*_, and *f*_*αβ*_ are the volume fractions of the glassy matrix (*α*), B2 CuZr crystals (*β*) larger than 250 μm, and the mixed (*α + β*) constitute consisting the remaining B2 CuZr crystals and the amorphous phase in a small scale, respectively^10^,^17^. Furthermore, the 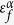, 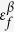, and 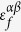 are the fracture strains of the *α, β*, and (*α + β)* constitutes, respectively. *K* is a dimensionless constant which accounts for the constraint effect of the *α* and/or *β* on the *α + β*. It is seen that the fracture strain of CuZr-based BMG composites, which also can be used to evaluate the plastic strain since the elastic strain of BMG composites is almost constant (i.e. about 2%)^8^,^10^,^17^, strongly depends on the proportion of the (*α* + *β*) constitute and the *K* constant if the total crystalline volume fraction *f*_*β*_ is fixed. In our case, the zone B mainly consists of the B2 CuZr crystals and the amorphous phase in a small scale (Figs 3 and 5a), whichshould belong to the (*α* + *β*) constitute. Compared with previous results^8^,^10^,^17^, the volume fraction of the (*α* + *β*) constitute is quite larger in the present BMG composites. As a result, these interdispersed microstructures could lead to significantly improved ductility for the present BMGs composites.

**Figure 6:**
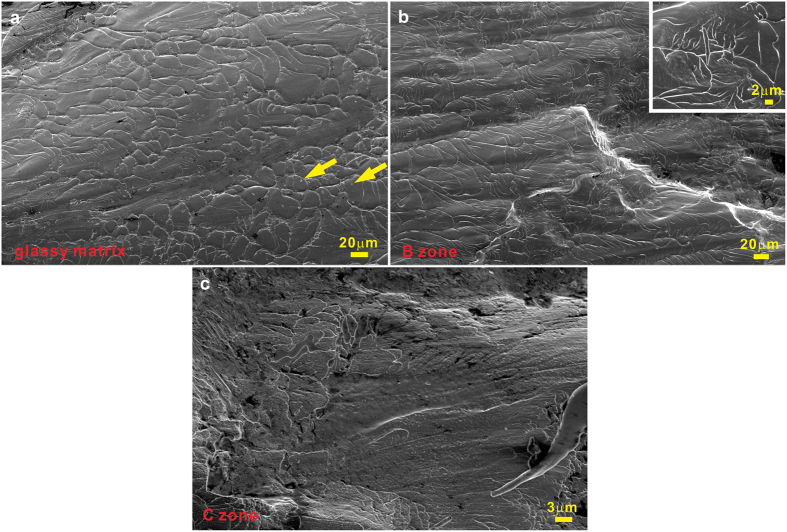
The fracture surfaces of (**a**) the glassy matrix, (**b**) the heat-affected zone, and (**c**) the molten zone for the locally melting samples after deformation.

The deformed samples exhibit different and complicated fracture angles depending on the local melting treatment and volume fraction of crystallites (Fig. 4b–d). There are also some common fracture features observed in different samples (Figs 5 and 6). First of all, there are multiple shear bands in the glassy matrix (Fig. 5b), being similar to those in BMGs composites reported in Refs 4,5,7,8,12, 13, 14, 15, 16, 17, 18, 19, 20. Some finer and closely woven shear bands and martensitic plates can be observed in the heat-affected zone B (Figs 4b–d and 5b), further confirming the existence of amorphous phase as discussed above. For the molten zone A, obvious martensitic plates are observed, indicating the occurrence of martensitic transformation upon loading (Fig. 5c)^4^,^5^,^7^,^8^,^12^,^13^,^14^,^15^,^16^,^17^,^18^,^19^,^20^. It has been shown that the elastic energy stored in a BMG sample - testing machine system is mainly consumed by the initiation and propagation of shear bands during deformation^44^. For CuZr-based BMG composites, the martensitic transformation can also dissipate a part of the stored elastic energy^45^ in the form of heat and the newly-generated interfacial free energy etc. Therefore, during plastic deformation of CuZr-based BMG composites, the total dissipated elastic energy by shear bands would be decreased. Furthermore, compared with BMGs, it becomes easier for multiple shear bands to form at the interface between the crystals and the glassy matrix in the BMG composites due to their large elastic mismatch^46^ (see Figs 3(b) and 5(c)). Based on the conservation of energy for the sample-machine system, even though the shear bands can operate more easily, less elastic energy would be consumed for the subsequent propagation of multiple shear bands. Besides, a certain amount of B2 CuZr crystals which locate at the driven path of the dominant shear band could directly block the rapid propagation of the dominant shear band to some extent. As a result, an enhancement of the shear banding stability of BMG composites can be reached.

Furthermore, the facture features also provide important information on dynamic fracture process, which strongly depends on ductility, toughness, deformation rate, and fracture mode^47^,^48^,^49^. Usually, the formation of the vein-like patterns is closely linked to the significant softening or reduced viscosity within shear bands when the final fracture occurs^47^. The origin responsible for the softening could be largely attributed to shear-induced structural disordering or temperature rise^48^,^49^. Even some liquid-like features such as molten droplets can be observed in the vein-like structures (see the yellow arrows in Fig. 6a). For the present BMG composites, the fracture surface of the glassy matrix is dominated by the vein-like patterns (Fig. 6a). Generally, the vein-like patterns slightly change into the river-like patterns which is close to striation patterns in the edge of the fracture surface^49^. However, in our case, the river-like patterns not only appear in the edge of the fracture surface but also in the zone B. As shown in Fig. 6b, the fractography of the zone B mainly exhibits the river-like patterns including some granular structures, implying that the interdispersed microstructures could affect the softening within shear bands. For the fracture surface of the zone A, a large amount of granular structures can be observed (Fig. 6c). This indicates that the presence of B2 crystals can also effectively change the fracture of the glassy matrix. As has been demonstrated earlier^4^,^5^,^6^,^7^,^8^,^12^,^13^,^14^,^15^,^16^,^17^,^18^,^19^,^20^, the deformation of the CuZr-based BMG composites not only depends on the multiplication of shear bands and the occurrence of martensitic transformation but also on the complicated stress states in the glassy matrix. In our case, when the B2 crystals locate nearby or at the main shearing direction (Fig. 4b and d), the stress distribution in the glassy matrix should be distorted, severely suppressing the rapid propagation of main shear bands and the subsequent formation of micro-cracks. This yields CuZr-based BMG composites with better mechanical properties.

## Conclusions

In summary, a new strategy based on local melting of BMG to fabricate strong and plastically deformable CuZr-based BMG composites was introduced. During local and fast melting process, the BMGs can be locally melt and heated together with the homogeneity of the glassy matrix, leading to the precipitation of B2 CuZr crystals in the glassy matrix. By controlling the local melting process, the size, volume fraction, and distribution of B2 CuZr crystals can be artificially adjusted. This enables effective suppressing the propagation of multiple shear bands in the glassy matrix and as a consequence to enhance mechanical properties of CuZr-based BMG composites, particularly to improve their plasticity and toughness.

## Methods

Cu_47_Zr_47_Al_6_ master alloys were prepared by arc-melting appropriate amounts of the constituting elements (purity 99.9%). In order to ensure their chemical homogeneity, the master alloys were re-melted three times under a Ti-gettered argon atmosphere. The BMG plates with a cross-section of 1.5 mm × 10 mm and the length of about 80 mm were fabricated via suction-casting. Their amorphous structures were characterized using the X-ray diffraction (XRD, Rigaku D/max-rB) in reflection geometry. During local arc melting, the plates were placed on a water-cooled copper support. The time for each local melting was about 1 s. After the samples were cooled to room temperature, the melting procedure was started at a next point. The input power during the local melting was adjusted by controlling the electric current and voltage. The applied input powers of 317 ± 57 W, 422 ± 58 W, and 604 ± 124 W correspond to the currents/voltages of 15 ± 2 A/20 ± 2 V, 30 ± 2 A/14 ± 1 V, and 50 ± 2 A/12 ± 2 V, respectively. The structure of the samples before and after local melting were characterized by an XRD, an optical microscopy (OM, Carl Zeiss) and a scanning electron microscopy (SEM, Gemini 1530 and SU-70). The mechanical properties of samples were determined using a Vickers hardness tester (SCTMC MHV-1000Z), a nanoindenter (Anton Paar CSM-NHT2), and a universal mechanical testing machine (MTS CMT5305). The maximum applied force in the nanoindentation tests was 50 mN, the loading rate was 100 mN/min, and the holding time at the maximum applied force was 10 s. For the compression tests, the initial strain rate was 2.5 × 10^−4^ s^−1^, and the geometric size of the compression sampled was 1.5 mm × 1.5 mm × 3 mm.

## Additional Information

**How to cite this article**: Qin, Y.-S. *et al*. Local melting to design strong and plastically deformable bulk metallic glass composites. *Sci. Rep.* **7**, 42518; doi: 10.1038/srep42518 (2017).

**Publisher's note:** Springer Nature remains neutral with regard to jurisdictional claims in published maps and institutional affiliations.

## Supplementary Material

Supplementary Information
