# Supplementary material for: Local melting to design strong and plastically deformable bulk metallic glass composites
Source: Sci Rep. 2017 Feb 13;7:42518. doi: 10.1038/srep42518 (PMC5304197; doi:10.1038/srep42518)
Supplement: Supplementary Information [file srep42518-s1.pdf]

## SUPPLEMENTARY INFORMATION FOR

# Local melting to design strong and plastically deformable bulk metallic glass composites

Yue-Sheng Qin [1], Xiao-Liang Han [1], Kai-Kai Song [1, †], Yu-Hao Tian [1], Chuan-Xiao Peng [1], Li Wang [1, †], Bao-An Sun [2], Gang Wang [3], Ivan Kaban [4], and Jürgen Eckert [5, 6]

[1] School of Mechanical, Electrical & Information Engineering, Shandong University (Weihai), Wenhua Xilu 180, 264209 Weihai, P.R. China. [2] Centre for Advanced Structural Materials, Department of Mechanical and Biomedical Engineering, City University of Hong Kong, 999077 Hong Kong SAR, P.R. China. [3] Laboratory for Microstructures, Shanghai University, 200444 Shanghai, P.R. China. [4] IFW Dresden, Institute for Complex Materials, Helmholtzstraße 20, 01069 Dresden, Germany. [5] Erich Schmid Institute of Materials Science, Austrian Academy of Sciences, Jahnstraße 12, A-8700 Leoben, Austria. [6] Department Materials Physics, Montanuniversität Leoben, Jahnstraße 12, A-8700 Leoben, Austria. [†] Correspondence and requests for materials should be addressed to K.S. (email: songkaikai8297@gmail.com) or to L.W. (email: wanglihxf@sdu.edu.cn).

**This file includes:**

Supplementary Figure S1;

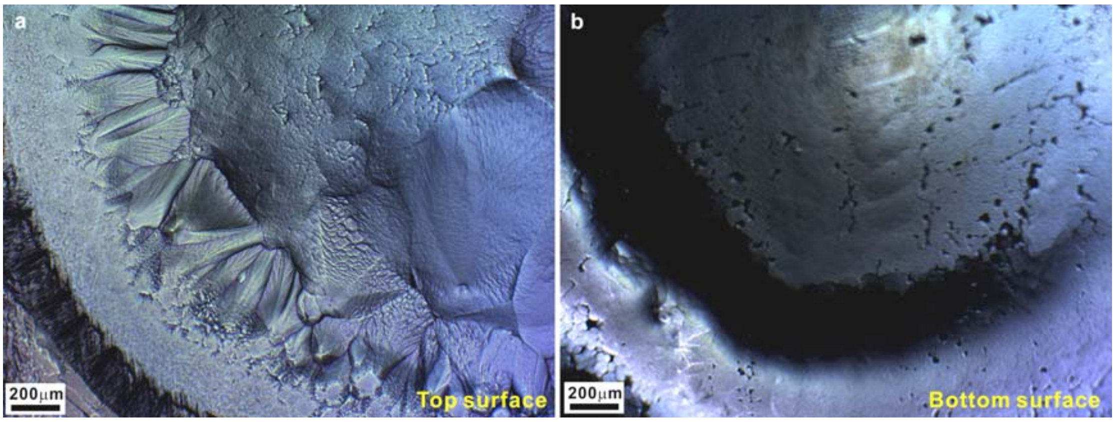


**Figure S1** The OM images taken from the top and bottom surfaces of a $Cu_{47}Zr_{47}Al_6$ plate after local melting with an input power of 604 ± 124 W.
